# Detection of *PRKAR1A* gene mutations in sporadic cardiac myxomas: a study of 24 cases

**DOI:** 10.1007/s00428-025-04049-x

**Published:** 2025-02-18

**Authors:** Elise Bekers, Diede A. G. van Bladel, Madeleine R. Berendsen, Astrid Eijkelenboom, J. Han J. M. van Krieken, Marc Ooft, Emiel Ruijter, Ad Verhagen, Uta E. Flucke, Blanca Scheijen

**Affiliations:** 1 Department of Pathology, Radboud University Medical Center, Nijmegen, The Netherlands; 2 Department of Pathology, The Netherlands Cancer Institute, Amsterdam, The Netherlands; 3 Princess Máxima Center for Pediatric Oncology, Utrecht, The Netherlands; 4 Pathology-DNA, Rijnstate Hospital, Arnhem, The Netherlands; 5 Department of Cardio Thoracic Surgery, Radboud University Medical Center, Nijmegen, The Netherlands

**Keywords:** Soft tissue tumor, Cardiac myxoma, *PRKAR1A*, Next-generation sequencing, Mutation analysis

## Abstract

The benign neoplasm cardiac myxoma represents one of the hallmarks of Carney complex (CNC), a familial multiple neoplasia syndrome. About 80% of the index cases have germline mutations in *PRKAR1A* encoding the RIα regulatory subunit of cAMP-dependent protein kinase A (PKA). However, the role of *PRKAR1A* gene mutations in the pathogenesis of non-CNC-associated sporadic cardiac myxoma is less well established. Here, we investigated the presence of *PRKAR1A* gene variants in a cohort of 24 sporadic cardiac myxomas using targeted next-generation sequencing. Our study shows that 14 out of 24 cases (58%) harbor *PRKAR1A* gene mutations, represented mostly by frameshift, nonsense, and splice site mutations (together 84%), leading to a premature stop codon predicted to be degraded via non-sense mediated mRNA decay. The other 16% of *PRKAR1A* genetic alterations involved missense mutations, often located in important functional domains of the regulatory subunit RIα. Notably, 64% (*n* = 9/14) of the cases harbored more than one *PRKAR1A* gene variant, suggesting compound heterozygous mutations either in *cis* or *trans*. In conclusion, *PRKAR1A* gene mutations associated with loss of RIα function leading to increased PKA activity were observed in ~ 60% of sporadic cardiac myxomas, strongly supporting an essential role for PKA in mediating formation of cardiac myxoma.

**Supplementary Information:**

The online version contains supplementary material available at 10.1007/s00428-025-04049-x.

## Introduction

Cardiac myxoma is the most common primary tumor of the heart, manifesting itself mainly in middle-aged patients between the third and sixth decades of life, and affects woman on average twice more than men [1]. It is a benign mesenchymal neoplasm composed of stellate, ovoid, or plump spindle cells in a myxoid stroma with prominent vascularization [2, 3]. Most tumors occur sporadic, but up to 10% arise as a component of the Carney complex (CNC), a multiple neoplasia syndrome characterized by cardiac and extracardiac myxomas, mucocutaneous pigmentation, endocrine hyperactivity due to tumors in the corresponding organs, and malignant melanotic nerve sheath tumor [4, 5]. Cardiac myxomas represent the most common non-cutaneous lesions in CNC (in 20–40% of the patients), which can arise anywhere in the heart, and are often multiple and recurrent. In contrast, sporadic cardiac myxomas develop almost exclusively in the left atrium and are generally solitary without recurrence after standard surgical treatment [6]. Approximately 70% of the CNC patients show a familial history, while the remaining patients develop tumors as a result of a de novo germline defect. Up to 80% of CNC lesions are caused by genetic alterations affecting the *PRKAR1A* gene, located on chromosome 17q22-24 [7–10]. These contain mostly exonic and splice site mutations, while larger deletions, including those affecting promoter/enhancer regions*,* have been reported in only a few cases [11–14].

*PRKAR1A* is a tumor suppressor gene encoding for the regulatory subunit type 1 alpha (RIα) of the 3′,5′-cyclic adenosine monophosphate (cAMP)-dependent protein kinase A (PKA) enzyme, which is constitutively expressed in all cell types [15]. In its inactive state, PKA is an hetero-tetramer comprised of a regulatory subunit dimer (type I subunits: RIα, RIβ; type II subunits: RIIα, RIIβ) and two catalytic subunits (three different forms: Cα, Cβ,PRKX) [16, 17]. RIα sequesters the PKA catalytic subunits and inhibits PKA kinase activation in the absence of second messenger cAMP. Mutations within the *PRKAR1A* gene result in a mutant protein or lead to non-sense mRNA lacking translation into RI $\alpha$ protein, also called non-sense mediated mRNA decay [10, 18]. This loss of RI $\alpha$ function eventually results in unrestrained activity of the PKA catalytic subunits leading to increased cell proliferation and tumor formation.

Initial reports failed to identify *PRKAR1A* mutations, loss of heterozygosity, or microsatellite instability in sporadic non-syndromic cardiac myxomas [19–21]. However, in three other studies employing predominantly, Sanger sequencing *PRKAR1A* variants were detected in 13 out of 19 (68%) [22], 9 of 29 (31%) [23], and 39 of 61 (64%) of such lesions [24] and in an individual case [25]. A complicating factor for mutation detection in cardiac myxomas is the relative hypocellularity, where low concentrations of genomic DNA are isolated. Therefore, genetic analysis of this tumor type will benefit from robust and sensitive techniques for mutation detection using deep targeted next-generation sequencing (tNGS). In this study, we applied two different complementary tNGS-based approaches for reliable *PRKAR1A* mutation detection in a cohort of 24 sporadic cardiac myxomas.

## Materials and methods

### Patient cohort and tissue specimens

A total of 24 cases of sporadic cardiac myxoma were retrieved from the archives of the Pathology Departments of the Radboud University Medical Center (Nijmegen, The Netherlands) and Rijnstate Hospital (Arnhem, The Netherlands). Patients were diagnosed between 2000 and 2016 and showed no signs of CNC (multiple myxomas, spotty skin pigmentation, endocrine overactivity with corresponding lesions). Histopathological review of the H&E-stained slides ($4-\mu \text{m}$ formalin-fixed and paraffin-embedded (FFPE) tissue) was performed according to the 2021 World Health Organization criteria [26]. All samples and clinical information were collected in accordance with the Declaration of Helsinki and of Taipei.

### Genomic DNA isolation

Three 20-µm sections were cut from each specimen of FFPE tissue and digested at 56 °C for at least 1 h in the presence of TET-lysis buffer (10 mmol/L Tris/HCl pH8.5, 1 mmol/L EDTA pH8.0, 0.01% Tween-20) with 5% Chelex-100 (Bio-Rad, Hercules, CA, USA), 15 μg/mL GlycoBlue (Thermo Fisher Scientific, Waltham, MA, USA), and 400 μg proteinase K (Qiagen, Hilden, Germany), followed by inactivation at 95 °C for 10 min and centrifugation for 10 min at 20,000 × g. Supernatant containing genomic DNA was purified with QIAamp DNA Micro Kit (Qiagen). Alternatively, QIAamp DNA FFPE Tissue Kit (Qiagen) was used directly for genomic DNA isolation according to manufacturer’s protocol. DNA concentration was measured with Qubit Broad Range Kit (Thermo Fisher Scientific).

### PRKAR1A mutation analysis by next-generation sequencing

For the detection of *PRKAR1A* gene mutations, two different targeted next-generation sequencing (tNGS) approaches were employed: (i) Ion Torrent amplicon-NGS (Ion AmpliSeq) and (ii) single molecule molecular inversion probe (smMIP) technique followed by paired-end sequencing on Illumina NovaSeq platform (Illumina, San Diego, CA, USA). For the Ion AmpliSeq method, multiplex PCR was performed with 20 ng input DNA to amplify the intron–exon boundaries and protein-coding genomic regions of exons 2–11 of the *PRKAR1A* gene (Supplementary Table 1). After beads purification of the amplicons (Agencourt AMPure XP Beads, Beckman Coulter, Brea, CA, USA), library preparation, and amplification (Ion Plus Fragment Library kit and Ion Xpress Barcode Adapters kit, Thermo Fisher Scientific) as described previously [27], the samples were pooled with equal DNA concentrations for sequencing on Ion Torrent PGM using Ion 318™ Chip Kit v2 BC (Ion Torrent™, Thermo Fisher Scientific). An automated workflow for smMIP pool and library preparation was performed on 100 ng input DNA for each sample as described previously [28], and custom molecular inversion probes (MIPs) targeting the intron–exon boundaries and protein-coding genomic regions of exons 2–11 of *PRKAR1A* (Supplementary Table 2) were included in a larger smMIP panel as reported elsewhere [29]. NGS data output was analyzed with SeqNext software (JSI Medical Systems, Ettenheim, Germany) with a minimum coverage set at *n* = 5 total variant reads for each single nucleotide variant identified by Ion AmpliSeq (without the ability to distinguish PCR duplicates) or MIP (eliminating PCR duplicates after consensus clustering through identification of the unique molecular identifier) and a minimum variant allele frequency (VAF) of 5% for both Ion AmpliSeq and smMIP NGS datasets (minimum read depth: 100 ×). For mutational calling, synonymous variants, intronic variants (with exception of splice sites), and single nucleotide polymorphisms (SNPs) related to known germline variants were excluded.

## Results

### Clinical characteristics

The 24 cardiac myxomas analyzed were from 14 females (58%) and 10 males (42%) with a median age of 63 years (range 35–86 years), who presented without any other clinical manifestations of CNC (CM01-CM24; Table 1). All tumors but one were located in the left atrium. In one case (CM16), the tumor was spanning both, left and right cardiac chambers. All samples were tumor resection specimens. Besides surgery, no additional treatment was given. During follow-up (range 7–23 years), there were no further events.

**Table 1:** Clinical characteristic patient cohort sporadic cardiac myxoma

Case number	Gender	Age at diagnosis	Location cardiac myxoma
CM01	F	54	Left atrium
CM02	F	77	Left atrium
CM03	F	72	Left atrium
CM04	F	74	Left atrium
CM05	M	66	Left atrium
CM06	F	64	Left atrium
CM07	F	38	Left atrium
CM08	M	71	Left atrium
CM09	M	62	Left atrium
CM10	M	69	Left atrium
CM11	M	63	Left atrium
CM12	F	36	Left atrium
CM13	M	39	Left atrium
CM14	F	42	Left atrium
CM15	M	55	Left atrium
CM16	M	68	Left and right atrium
CM17	F	57	Left atrium
CM18	F	73	Left atrium
CM19	M	53	Left atrium
CM20	F	57	Left atrium
CM21	F	43	Left atrium
CM22	F	86	Left atrium
CM23	M	75	Left atrium
CM24	F	35	Left atrium

### Histopathology

**Fig. 1:**
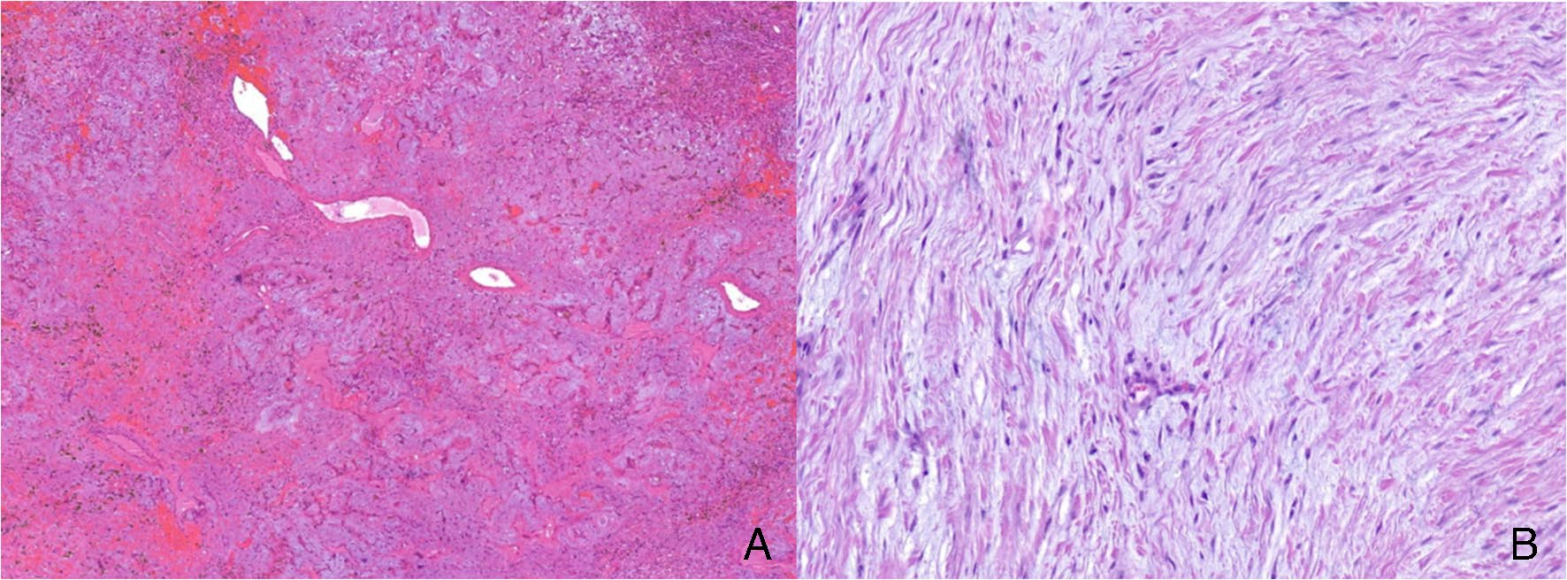
**A** Hematoxylin and eosin (H&E) staining shows the characteristic morphology of cardiac myxoma (case 13) with a prominent vasculature in a myxoid matrix combined with inflammatory cells and hemorrhage with hemosiderin deposits.** B** High power view of case 18 demonstrating polygonal/stellate lesional cells with an oval nucleus with open chromatin and indistinct nucleoli and an eosinophilic cytoplasm, set in an abundant myxoid background

All cases showed the characteristic features of a cardiac myxoma, represented by sparsely distributed cytological bland-looking spindle and stellate-shaped or plump cells with indistinct cell borders embedded in a myxoid matrix with prominent vascularity and signs of hemorrhage (Fig. 1).

## *PRKAR1A* gene mutation analysis by two different next-generation sequencing approaches

**Fig. 2:**
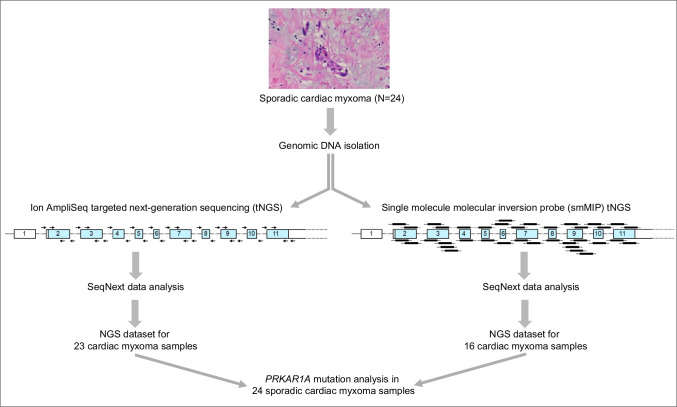
Schematic overview of the targeted next-generation sequencing approaches to detect *PRKAR1A* gene mutations in sporadic cardiac myxoma. On the left side, the workflow of the Ion AmpliSeq method and, on the right side, the single molecule molecular inversion probe (smMIP) approach is shown. The relative positions of the sense primers and smMIPs have been mapped above the *PRKAR1A* exons, while antisense primers and smMIPs are indicated below the *PRKAR1A* exons

In all cases, sufficient FFPE tissue was available for genomic DNA isolation and molecular analysis, focusing on *PRKAR1A* mutation analysis without additional copy number variation analysis. Two different complementary tNGS approaches were used (Fig. 2). First, a custom Ion AmpliSeq method was designed, where the protein-coding sequence and intron–exon junctions of exons 2–11 of *PRKAR1A* were amplified with oligonucleotide primers generating amplicons ranging in size from 140 to 217 nucleotides, which were prepared for sequencing on Ion Torrent platform. Secondly, the smMIP technology was used that targets genomic regions of 113 nucleotides in size, which we previously employed as a successful approach to identify novel *GNAS* mutations in intramuscular myxoma [31]. Important advantages of smMIP compared to Ion AmpliSeq relates to ability to identify PCR artifacts by the inclusion of a unique molecular identifier (UMI) sequence of (in our case) 8 random bases in the ligation probe representing 4^8^ = 65,536 single molecule possibilities. Since every smMIP molecule with an individual UMI-tag can only hybridize to one genomic DNA fragment, the UMI sequence is able to correct for PCR duplicates in the bioinformatic analysis pipeline. Furthermore, the smMIPs are designed to capture overlapping regions on both sense and antisense DNA strands, thereby distinguishing FFPE artifacts related to single strand cytosine deamination variants from actual gene mutations detected on both DNA strands.

## Identification of *PRKAR1A* gene mutations in sporadic cardiac myxoma

Each case was subjected to both tNGS approaches involving Ion AmpliSeq and smMIP-based mutation analysis of the *PRKAR1A* gene. Based on the threshold settings employed for mutation detection (see “Materials and Methods” section), one sample (CM05) was excluded from the data analysis after Ion AmpliSeq tNGS, while eight samples (CM04, CM06, CM09, CM10, CM12, CM13, CM14, CM22) did not fulfill the criteria for *PRKAR1A* mutation detection using the smMIP approach (“No data” in Supplementary Table 3). However, for all 24 cases, at least one tNGS approach provided reliable NGS datasets to investigate the presence of *PRKAR1A* gene mutations (Supplementary Table 3). Samples analyzed by both tNGS approaches showed identical results, except for one sample (CM16), where only Ion AmpliSeq detected a *PRKAR1A* variant (c.955G > A; p.Gly319Arg), but not the smMIP method.

**Fig. 3:**
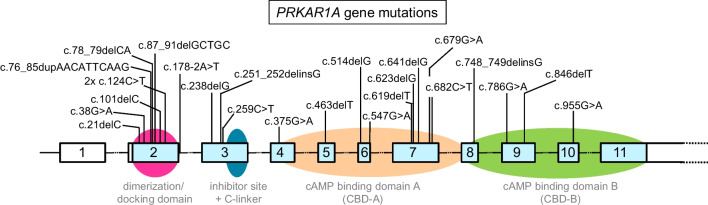
Summary of the identified *PRKAR1A* gene mutations (*n* = 25) in sporadic cardiac myxoma. The different exonic regions (exons 1–11) of the human *PRKAR1A* gene are shown, and coding regions are indicated in blue. The functional protein domains of the regulatory subunit RI alpha that are encoded by the different *PRKAR1A* exons are represented by the colored circles

Combining the results from both tNGS approaches, *PRKAR1A* variants were identified in 14 of 24 (58%) sporadic cardiac myxomas (Table 2). These involved a total of 25 distinct variants within the *PRKAR1A* gene, including 56% (*n* = 14/25) frameshift mutations, 24% (*n* = 6/25) missense mutations yielding amino acid substitutions, 16% (*n* = 4/25) nonsense mutations resulting in a premature stop codon, and 4% (*n* = 1/25) splice site mutation (Fig. 3). The frameshift mutations resulted from indels with the deletion and/or insertion of single nucleotides (*n* = 11/14; 79%) or multiple nucleotides (*n* = 3/14; 21%). The *PRKAR1A* mutations were distributed over all coding exons, except exon 11. The majority of variants were located in exon 2 (c.1–c.177) and exon 7 (c.550–c.708). Loss-of-function mutations resulting from frameshift and nonsense mutations together with the splice site mutation were classified as pathogenic (*n* = 11/25; 44%) or likely pathogenic (*n* = 8/25; 32%), while the six missense mutations were categorized as variant of uncertain significance (*n* = 6/25; 24%) (p.Arg13His located in the dimerization/docking domain; p.Met125Ile, p.Asp183Asn, p.Asp227Asn located in c-AMP binding domain A; p.Gly319Arg located in c-AMP binding domain B; p.Pro87Ser in the C-linker region). In total, 11 out of 25 variants (44%) were previously identified in CNC patients, 2 additional variants detected in unrelated sporadic cardiac myxoma cases, while 12 variants represented novel genetic alterations in *PRKAR1A* (Supplementary Table 3). The median VAF of all identified variants was 17% (range 6–38%) for Ion AmpliSeq and 24% (range 14–44%) for smMIP, indicating that these single nucleotide variants reflected somatic mutations. Notably, 9 out of 14 (64%) cases showed more than one *PRKAR1A* variant, suggestive for the presence of compound heterozygous *PRKAR1A* mutations in sporadic cardiac myxoma. In one case (CM09), this involved two missense mutations (c.259C > T, c.679G > A), while in the other eight cases, these represented a combination of either a frameshift and/or nonsense mutations.

**Table 2:** *PRKAR1A* gene mutation analysis in sporadic cardiac myxoma

Case number	DNA variant *PRKAR1A*	Protein alteration PRKAR1A	VAF Ion AmpliSeq	VAF smMIP	Effect
**CM01**	c.238delG	p.Asp80Metfs*49	31%	24%	Frameshift
	c.682C > T	p.Arg228Ter	25%	25%	Nonsense
**CM02**	c.124C > T	p.Arg42Ter	20%	20%	Nonsense
	c.178-2A > T	p.?	17%	19%	Altered splicing
**CM03**	c.623delG	p.Gly208Glufs*14	26%	29%	Frameshift
	c.641delC	p.Thr214Metfs*8	21%	28%	Frameshift
**CM04**	c.87_91delGCTGC	p.Leu30Glnfs*13	12%	No data	Frameshift
	c.251_252delinsG	p.Pro84Argfs*45	12%	No data	Frameshift
**CM05**	No variant detected	-	No data	NVD†	-
**CM06**	No variant detected	-	NVD	No data	-
**CM07**	No variant detected	-	NVD	NVD	-
**CM08**	c.748_749delinsG	p.Leu250Valfs*7	6%	No data	Frameshift
**CM09**	c.259C > T	p.Pro87Ser	8%	No data	Missense
	c.679G > A	p.Asp227Asn	6%	No data	Missense
**CM10**	No variant detected	-	-	No data	-
**CM11**	c.101delC	p.Ser34Leufs*95	17%	73%^#^	Frameshift
**CM12**	c.786G > A	p.Trp262Ter	18%	No data	Nonsense
**CM13**	c.21delC	p.Ala8Profs*121	9%	No data	Frameshift
	c.78_79delCA	p.Ile27Serfs*17	11%	No data	Frameshift
**CM14**	No variant detected	-	NVD	No data	-
**CM15**	c.463delT	p.Ser155Argfs*10	16%	16%	Frameshift
**CM16**	c.955G > A	p.Gly319Arg	10%	NVD	Missense
**CM17**	No variant detected	-	NVD	NVD	-
**CM18**	c.514delG	p.Asp172Ilefs*5	20%	14%	Frameshift
	c.846delT	p.Ile282Metfs*15	17%	23%	Frameshift
**CM19**	No variant detected	-	NVD	NVD	-
**CM20**	No variant detected	-	NVD	NVD	-
**CM21**	No variant detected	-	NVD	NVD	-
**CM22**	c.124C > T	p.Arg42Ter	7%	No data	Nonsense
	c.375G > A	p.Met125Ile	7%	No data	Missense
	c.38G > A	p.Arg13His	15%	No data	Missense
	c.547G > A	p. Asp183Asn	26%	No data	Missense
**CM23**	No variant detected	-	NVD	NVD	-
**CM24**	c.76_85dupAACATTCAAG	p.Ala29Glufs*19	33%	44%	Frameshift
	c.619delT	p.Tyr207Metfs*15	38%	36%	Frameshift

*VAF*, variant allele frequency. No data: too few reads. †NVD: no variant detected.^#^Relative low sequencing depth at that position with smMIP may have resulted in a skewed VAF. This value was not included in the calculation of the median VAF

## Discussion

Cardiac myxomas are the most common primary tumors of the heart with an estimated prevalence of 0.03% in the general population [32]. The pathobiology of cardiac myxomas has still remained unclear so far, but there is evidence of an obvious role for *PRKAR1A* mutations in CNC-associated cardiac myxomas [5]. However, in sporadic lesions, in the beginning, it appeared more challenging to assess the role of *PRKAR1A* gene mutations. Sensitive methods involving tNGS are required to detect gene variants in hypocellular tissues at lower abundancy. Here, we presented two different tNGS methods for *PRKAR1A* gene mutation detection in 24 sporadic cardiac myxomas. One method involved Ion AmpliSeq where amplicons generated by multiplex PCR of *PRKAR1A* exonic regions were sequenced on Ion Torrent platform, while the second method used UMI-tagged MIPs to capture genomic fragments from both DNA strands of the *PRKAR1A* gene that were sequenced on an Illumina platform. Integration of both NGS datasets showed that 14 of 24 cases (58%) displayed *PRKAR1A* gene variants with an average median VAF of 21%, due to the relative low tumor percentage. This frequency is similar to the findings of two previous studies where *PRKAR1A* gene mutations were detected in 68% and 64% of sporadic cardiac myxomas, respectively [22, 24]. In the study of Maleszewski et al., they only found *PRKAR1A* gene mutations in 31% of cases. This could possibly be explained by their reported low quantity and quality of the DNA (only 26% was adequate) and the Sanger sequencing technique, which has lower sensitivity and limitations in detecting larger deletions or duplications [23].

The majority of variants that were identified by tNGS in our cases represented genetic alterations that are predicted to result in RIα loss of function, including 14 frameshift and 4 nonsense mutations, which as a single mutation most likely result in haploinsufficiency due to non-sense-mediated mRNA decay (NMD) or truncated protein resulting from a premature stop codon. These types of genetic alterations also predominate in CNC-associated *PRKAR1A* germline variants [10, 18, 20]. However, some *PRKAR1A* variants may escape NMD, especially those that cluster in cAMP binding domain A (CBD-A) [33], thereby giving rise to the expression of an altered and dysfunctional RI$\alpha$ protein. In addition, *PRKAR1A* mutations can also promote accelerated RIα protein degradation [34]. Similar to findings in other reported sporadic as well as CNC-associated cardiac myxomas [23, 25, 35], we furthermore identified missense mutations that mapped in the dimerization/docking domain or cAMP binding domains of RIα. Notably, mutation p.Met125Ile was located in the isoform-specific N3A motif representing the RIα homodimer interface [36] and mutation p.Asp227Asn in the hinge domain of CBD-A, both representing allosteric hotspots [37]. However, it still remains to be established whether each of the identified missense mutations will affect the RIα function. In addition, we identified one mutation outside the *PRKAR1A* coding sequencing, representing a splice site mutation affecting RIα function, also occurring frequently in CNC associated lesions [7, 10, 18].

Another important observation from our study is that almost two-thirds of the sporadic cardiac myxomas displayed multiple *PRKAR1A* variants, in line with previous findings [24, 25]. Although mosaicism could formerly not be excluded [38], it is very likely that the majority of these cases show *PRKAR1A* inactivation either in *cis* or *trans* by compound heterozygous mutations. Thus, the occurrence of cardiac myxoma is strongly associated with uncontrolled PKA activation, either through germline CNC-associated *PRKAR1A* mutation combined with loss-of-heterozygosity as seen in the other CNC-associated lesions [8] or somatic loss-of-function mutations. Heterozygous knock-out of the *PRKAR1A* gene in mice leads to extracardiac myxomatous soft tissue tumors [22], in line with some of the CNC-associated tumors including cardiac myxomas. However, mice displaying cardiac-specific heterozygous ablation of *PRKAR1A* show no signs of cardiac tumor formation but instead diminished cardiomyocyte hypertrophy and augmented cardiomyocyte necrosis [39, 40].

Cardiac myxomas negative for *PRKAR1A* mutation(s) may activate PKA through other molecular pathways. These include activating micro-insertions in *PRKACA* encoding the catalytic subunit Cα of PKA [41] and loss-of-function mutations in *KIF1C*, a member of the kinesin superfamily of molecular motor proteins, resulting in decreased *PRKAR1A* expression [42]. Other candidates that could affect PKA activity are regulators of RI, RII, and C subunits [17, 43], but specific mutations in their genes have not yet been linked to cardiac myxoma.

In conclusion, our findings indicate that about 60% of sporadic cardiac myxoma harbor *PRKAR1A* mutations leading most often to loss of the regulatory subunit RIα and unscheduled activation of the PKA enzyme. Notably, 64% of the affected tumors show multiple *PRKAR1A* variants suggesting *PRKAR1A* inactivation by compound heterozygous mutations. This is in concert with previously published results. Further investigation using genome-wide next-generation sequencing is required to investigate and identify other potential driver mutations involved in the pathogenesis of sporadic cardiac myxomas.

## Supplementary Information

Below is the link to the electronic supplementary material.

Supplementary file1 (XLSX 19 KB)

## Data Availability

Next-generation sequencing data files that support the findings of this study are available from the corresponding author upon reasonable request. Data is located in controlled access data storage at Radboud University Medical Center.
